# Association between dietary flavonol intake and mortality risk in the U.S. adults from NHANES database

**DOI:** 10.1038/s41598-024-55145-y

**Published:** 2024-02-25

**Authors:** Zhiqiang Zong, Xiang Cheng, Yang Yang, Jianchao Qiao, Jiqing Hao, Fanfan Li

**Affiliations:** 1 Department of Oncology, The Second Affiliated Hospital of Anhui Medical University, No. 678 Furong Road, Hefei, 230601 China; 2 Department of Clinical Medicine, The Second School of Clinical Medicine, Anhui Medical University, No. 81 Meishan Road, Hefei, 230032 China; 3 Department of Oncology, The First Affiliated Hospital of Anhui Medical University, No. 218 Jixi Road, Hefei, 230022 China

**Keywords:** Flavonol, Cancer, Alzheimer's disease, Cardiovascular diseases, All-cause mortality, Specific-cause mortality, National Health and Nutrition Examination Survey, Cancer, Cardiology, Diseases, Medical research, Oncology, Risk factors

## Abstract

Using updated National Health and Nutrition Examination Survey (NHANES) follow-up data, and a large nationwide representative sample of adult U.S. citizens, the aim of this study was to explore the relationship between dietary flavonol intake, all-cause and cause-specific mortality risks. In this prospective cohort study based on NHANES (2007–2008, 2009–2010, and 2017–2018), a total of 11,679 participants aged 20 years and above were evaluated. The amount and type of food taken during a 24-h dietary recall were used to estimate dietary flavonol intake, which includes total flavonol, isorhamnetin, kaempferol, myricetin, and quercetin. Each analysis of the weighted data was dealt with in accordance with the NHANES reporting requirements' intricate stratification design. The Cox proportional risk regression model or Fine and Gray competing risks regression model were applied to evaluate all-cause and cause-specific mortality risks, respectively. The follow-up period was calculated using the time interval between the baseline and the death date or December 31, 2019 (whichever occurs first). Each data analysis was performed between October 1, 2023, and October 22, 2023. Dietary flavonol intake included total flavonol, isorhamnetin, kaempferol, myricetin, and quercetin. Up to December 31, 2019, National Death Index (NDI) mortality data were used to calculate mortality from all causes as well as cause-specific causes. A total of 11,679 individuals, which represents 44,189,487 U.S. non-hospitalized citizens, were included in the study; of these participants, 49.78% were male (n = 5816), 50.22% were female (n = 5, 863); 47.56% were Non-Hispanic White (n = 5554), 18.91% were Non-Hispanic Black (n = 2209), 16.23% were Mexican American (n = 1895), and 17.30% were other ethnicity (n = 2021); The mean [SE] age of the sample was 46.93 [0.36] years, with a median follow-up of 7.80 years (interquartile range, 7.55–8.07 years). After adjusting covariates, Cox proportional hazards models and fine and gray competing risks regression models for specific-cause mortality demonstrated that total flavonol intake was associated with all-cause (HR 0.64, 95% CI 0.54–0.75), cancer-specific (HR 0.45, 95% CI 0.28–0.70) and CVD-specific (HR 0.67, 95% CI 0.47–0.96) mortality risks; isorhamnetin intake was associated with all-cause (HR 0.72, 95% CI 0.60–0.86), and cancer-specific (HR 0.62, 95% CI 0.46–0.83) mortality risks; kaempferol intake was associated with all-cause (HR 0.74, 95% CI 0.63–0.86), and cancer-specific (HR 0.62, 95% CI 0.40–0.97) mortality risks; myricetin intake was associated with all-cause (HR 0.77, 95% CI 0.67–0.88), AD-specific (HR 0.34, 95% CI 0.14–0.85), and CVD-specific (HR 0.61, 95% CI 0.47–0.80) mortality risks; quercetin intake was associated with all-cause (HR 0.66, 95% CI 0.54–0.81), cancer-specific (HR 0.54, 95% CI 0.35–0.84), and CVD-specific (HR 0.61, 95% CI 0.40–0.93) mortality risks; there was no correlation observed between dietary flavonol intake and DM-specific mortality. According to the current study, all-cause, AD, cancer, and CVD mortality risks declined with increased dietary flavonoid intake in the U.S. adults. This finding may be related to the anti-tumor, anti-inflammatory, and anti-oxidative stress properties of flavonol.

## Introduction

Biologically active polyphenolic chemicals called flavonoids are present in a wide variety of common vegetables, fruits, and other plant-based meals^1^. Flavonoids can be divided into six main subclasses, namely flavonol, isoflavones, flavanones, flavonoid glycosides, flavonolignans, and flavones. Of these, flavonol is the primary representative of the flavonoid subclasses; it is also the flavonoid that is found the most widely in nature and is thought to be the most active^1–3^. Additionally, the primary flavonol components of tea, onions, and berries are quercetin, kaempferol, myricetin, and isorhamnetin^4^. Flavonoids are widely present in our customary diet and may produce numerous advantageous biological processes in the body when consumed. Previous research has produced strong and consistent evidence that flavonoids may improve endothelial function and maintain and enhance nitric oxide (NO) status^5–7^. What's more, there's evidence that these substances can impact lipid and glucose metabolism, platelet function and thrombosis, inflammation, oxidative damage, blood pressure, and inflammation^8–10^. Furthermore, by specifically targeting important molecules and signaling pathways in a range of tumor cells, flavonoids can cause apoptosis and prevent cell growth and metastasis^11–14^. The discovery that food rich in flavonoids has cardioprotective and anti-tumor effects may be explained by these effects^15,16^.

Since 1999, the National Health and Nutrition Examination Survey (NHANES), which is population-based research, has been published every two years. It provides a nationwide typical sample of the U.S. non-institutional population^11^. The relationship between flavonol intake (i.e., total flavonol, isorhamnetin, kaempferol, myricetin, and quercetin) and cause-specific mortality (i.e., AD, cancer, CVD, and DM) in appropriate models has not been thoroughly reported in any literature, much less analyses that account for significant confounders and include trend chi-square tests for flavonoid intake and mortality. Based on the latest and comprehensive data from NHANES, we aim to thoroughly demonstrate the relationship between flavonoid intake and all-cause and cause-specific mortality risks, as well as its potential as an independent prognostic indicator for risk assessment of various diseases.

## Results

### Basic characteristic of including participants

In the 2007–2008, 2009–2010 and 2018–2019 circles of NHANES, the overall 11,679 participants aged 20 years and above were evaluated, representing 44,189,487 U.S. non-institutionalized residents. Table 1 listed the minimum, 25th percentile, median, mean, 75th percentile, and maximum of each flavonol intake. Table 2 provided an overview for the basic characteristics of included participants. The outcome presented that subjects in the highest category of total flavonol intake had higher proportion in groups with the following characteristics: males (1682 [55.84%] vs. 1238 [44.16%]), tended to be younger (≥ 80 y, 107 [2.15%] vs. 3.76 [15.72%]), Non-Hispanic White (1542 [74.6%] vs. 429 [6.78%]), married or living with a partner (1933 [67.95%] vs. 987 [32.05%]), educational attainment ≥ HS (2306 [86.84%] vs. 614 [13.16%]), at or above poverty line (2433 [89.63%] vs 487 [10.37%]), having alcohol consumption (2619 [92.80%] vs. 301 [7.20%]), BMI 18.5–30.0 (1785 [63.50%] vs. 40 [1.29%]), having history of DM (8467 [72.50%] vs. 3212 [27.5%]), hypertension (6718 [57.52%] vs. 4961 [42.48%]), hyperlipidemia (3104 [26.58%] vs. 8575 [73.42%]), congestive heart failure (11,352 [97.20%] vs. 327 [2.80%]), coronary heart disease (11,207 [95.96%] vs. 472 [4.04%]), angina (11,390 [97.53%] vs. 289 [2.47%]), heart attack (11,165 [95.60%] vs. 514 [4.40%]), and stroke (11,206 [95.95%] vs. 473 [4.05%]). Further, participants with intakes of isorhamnetin, kaempferol, myricetin, and quercetin also showed similar characteristics. More detailed information about basic characteristics of participants and dietary flavonol intake was presented in Table 2.

**Table 1:** Statistical description of dietary flavonol intakes.

Dietary intake of flavonol	Minimum (mg/day)	25th Percentile (mg/day)	Median (mg/day)	75th Percentile (mg/day)	Maximum (mg/day)
Total flavonol	0	5.81	12.51	23.99	288.14
Isorhamnetin	0	0	0.36	1.13	51.35
Kaempferol	0	0.53	2.09	6.18	110.93
Myricetin	0	0.19	0.51	1.6	79.76
Quercetin	0	3.36	7.74	15.07	196.51

**Table 2:** Sociodemographic, and Disease history characteristics of participants with dietary flavonol intake.

Characteristics	Study participants No. (%)	Total flavonol (mg/day)				Isorhamnetin (mg/day)				Kaempferol (mg/day)				Myricetin (mg/day)				Quercetin (mg/day)			
		Q1 [0, 5.81]	Q2 (5.81, 12.51]	Q3 (12.51, 23.99]	Q4 (23.99, 288.14]	Q1 [0, 0]	Q2 (0, 0.36]	Q3 (0.36, 1.13]	Q4 (1.13, 51.35]	Q1 [0, 0.53]	Q2 (0.53, 2.09]	Q3 (2.09, 6.18]	Q4 (6.18, 110.93]	Q1 [0, 0.19]	Q2 (0.19, 0.51]	Q3 (0.51, 1.6]	Q4 (1.6, 79.76]	Q1 [0, 3.36]	Q2 (3.36, 7.74]	Q3 (7.74, 15.07]	Q4 (15.07, 196.51]
Age, No. (%), years																					
20–29	1879 (16.09)	532 (22.65)	509 (20.70)	462 (18.11)	376 (15.72)	554 (21.64)	452 (19.45)	479 (19.63)	394 (15.80)	555 (22.84)	432 (17.70)	451 (18.72)	441 (17.60)	701 (29.21)	475 (19.62)	354 (14.92)	349 (14.08)	561 (23.96)	521 (21.62)	420 (16.41)	377 (15.23)
30–39	1942 (16.63)	489 (18.12)	457 (16.11)	500 (18.62)	496 (18.57)	488 (16.75)	450 (17.41)	487 (17.95)	517 (19.32)	478 (17.30)	489 (18.51)	454 (16.16)	521 (19.33)	565 (20.41)	490 (18.37)	454 (16.55)	433 (16.51)	502 (18.97)	484 (16.93)	466 (16.83)	490 (18.78)
40–49	2003 (17.15)	471 (19.63)	493 (18.93)	487 (19.27)	552 (19.50)	529 (19.86)	469 (19.43)	473 (17.81)	532 (20.15)	499 (19.64)	448 (17.98)	496 (19.16)	560 (20.32)	460 (18.18)	509 (20.00)	500 (19.83)	534 (19.26)	475 (19.52)	486 (18.23)	517 (20.93)	525 (18.67)
50–59	1912 (16.37)	417 (16.25)	450 (18.48)	502 (19.82)	543 (22.04)	476 (18.12)	413 (18.27)	494 (19.43)	529 (21.27)	438 (17.90)	446 (16.44)	471 (19.96)	557 (22.16)	397 (14.45)	453 (16.22)	514 (23.00)	548 (22.66)	444 (16.93)	437 (18.14)	489 (19.69)	542 (22.01)
60–69	2013 (17.24)	481 (11.49)	494 (13.95)	506 (13.19)	532 (14.18)	516 (11.96)	466 (12.74)	501 (14.34)	530 (14.04)	463 (11.83)	538 (15.64)	530 (13.10)	482 (12.67)	405 (9.24)	539 (14.02)	507 (13.87)	562 (15.42)	469 (10.46)	471 (13.43)	534 (14.04)	539 (14.80)
70–79	1275 (10.92)	341 (7.70)	324 (7.64)	296 (7.18)	314 (7.84)	352 (7.65)	315 (8.64)	312 (7.43)	296 (6.78)	335 (6.84)	344 (8.82)	331 (8.70)	265 (6.24)	264 (5.51)	329 (7.55)	338 (8.14)	344 (8.88)	303 (6.75)	331 (7.49)	310 (7.88)	331 (8.15)
≥ 80	655 (5.61)	191 (4.17)	193 (4.18)	164 (3.80)	107 (2.15)	208 (4.03)	169 (4.07)	155 (3.40)	123 (2.64)	166 (3.66)	209 (4.92)	190 (4.21)	90 (1.68)	146 (3.01)	188 (4.22)	172 (3.70)	149 (3.19)	165 (3.41)	194 (4.17)	182 (4.22)	114 (2.37)
*P* value	< 0.001	< 0.001				0.004				< 0.001				< 0.001				< 0.001			
Sex, No. (%)																					
Male	5816 (49.78)	1251 (40.78)	1378 (47.22)	1505 (51.35)	1682 (55.84)	1608 (50.33)	1200 (43.84)	1374 (47.12)	1634 (54.75)	1319(44.44)	1252 (42.46)	1471 (48.24)	1774 (59.29)	1299 (44.47)	1440 (46.49)	1617 (56.20)	1460 (49.16)	1375 (46.25)	1404 (47.24)	1421 (49.02)	1616 (53.65)
Female	5863 (50.22)	1671 (59.22)	1542 (52.78)	1412 (48.65)	1238 (44.16)	1515 (49.67)	1534 (56.16)	1527 (52.88)	1287 (45.25)	1615(55.56)	1654 (57.54)	1452 (51.76)	1142 (40.71)	1639 (55.53)	1543 (53.51)	1222 (43.80)	1459 (50.84)	1544 (53.75)	1520 (52.76)	1497 (50.98)	1302 (46.35)
*P* value	0.046	< 0.001				< 0.001				< 0.001				< 0.001				< 0.001			
Race/Ethnicity, No. (%)																					
Non-Hispanic White	5554 (47.56)	1314 (67.57)	1345 (69.05)	1353 (69.30)	1542 (74.76)	1531 (70.84)	1305 (70.85)	1342 (68.95)	1376 (70.77)	1319 (67.14)	1312 (68.07)	1368 (69.75)	1555 (75.35)	1168 (62.69)	1327 (67.77)	1463 (74.15)	1596 (75.55)	1273 (66.69)	1412 (70.42)	1354 (69.63)	1515 (74.13)
Non-Hispanic Black	2209 (18.91)	675 (13.58)	564(10.61)	541 (9.78)	429 (6.78)	768 (13.19)	561 (10.99)	491(9.35)	389 (6.58)	685 (13.89)	446 (8.71)	565 (9.88)	513 (8.05)	796 (16.11)	572 (10.65)	359 (6.23)	482 (7.81)	711 (14.06)	548 (10.41)	537 (9.29)	413 (6.87)
Mexican American	1895 (16.23)	464 (8.12)	490 (8.58)	506 (8.66)	435 (7.25)	344 (5.71)	413 (7.45)	522 (8.99)	616 (10.35)	448 (7.97)	632 (10.84)	434 (7.64)	381 (6.50)	488 (9.12)	544 (9.56)	540 (9.09)	323 (5.21)	459 (8.24)	472 (8.35)	486 (8.12)	478 (7.84)
Other	2021 (17.30)	469 (10.73)	521 (11.76)	517 (12.25)	514 (11.21)	480 (10.26)	455 (10.71)	546 (12.72)	540 (12.30)	482 (11.00)	516 (12.39)	556 (12.73)	467 (10.10)	486 (12.09)	540 (12.01)	477 (10.52)	518 (11.44)	476 (11.02)	492 (10.82)	541 (12.96)	512 (11.16)
*P* value	0.004	< 0.001				< 0.001				< 0.001				< 0.001				< 0.001			
Marital status, No. (%)																					
Unmarried or other	4569 (39.12)	1307 (41.02)	1162 (35.66)	1113 (34.88)	987 (32.05)	1385 (39.46)	1113 (38.38)	1126 (34.75)	945 (30.35)	1304 (41.01)	1095 (33.37)	1125 (36.20)	1045 (32.76)	1361 (42.62)	1166 (36.16)	1013 (33.22)	1029 (31.70)	1303 (41.31)	1202 (37.65)	1110 (34.70)	954 (29.96)
Married or living with a partner	7110 (60.88)	1615 (58.98)	1758 (64.34)	1804 (65.12)	1933 (67.95)	1738 (60.54)	1621 (61.62)	1775 (65.25)	1976 (69.65)	1630 (58.99)	1811 (66.63)	1798 (63.80)	1871 (67.24)	1577 (57.38)	1817 (63.84)	1826 (66.78)	1890 (68.30)	1616 (58.69)	1722 (62.35)	1808 (65.30)	1964 (70.04)
*P* value	0.002	< 0.001				< 0.001				< 0.001				< 0.001				< 0.001			
< HS	2887 (24.72)	860 (19.06)	725 (15.31)	688 (13.92)	614 (13.16)	839 (17.36)	645 (13.84)	712 (15.29)	691 (14.20)	856 (18.47)	781 (17.15)	645 (13.35)	605 (12.66)	832 (18.65)	791 (16.09)	720 (15.36)	544 (11.49)	892 (19.79)	700 (14.96)	683 (13.30)	612 (13.35)
≥ HS	8792 (75.28)	2062 (80.94)	2195 (84.69)	2229 (86.08)	2306 (86.84)	2284 (82.64)	2089 (86.16)	2189 (84.71)	2230 (85.80)	2078(81.53)	2125(82.85)	2278 (86.65)	2311 (87.34)	2106 (81.35)	2192 (83.91)	2119 (84.64)	2375 (88.51)	2027 (80.21)	2224 (85.04)	2235 (86.70)	2306 (86.65)
*P* value	0.005	< 0.001				0.005				< 0.001				< 0.001				< 0.001			
Poverty income ratio, No. (%)																					
Below poverty line (< 1.00)	2270 (19.44)	693 (16.07)	581(13.06)	509 (11.62)	487 (10.37)	708 (15.21)	517 (12.51)	546 (12.06)	499 (10.70)	687 (15.92)	572(13.24)	515 (11.29)	496 (10.68)	711 (17.23)	576 (12.45)	530 (11.84)	453 (9.71)	724 (16.95)	578 (13.41)	495 (10.46)	473 (10.30)
At or above poverty line (≥ 1.00)	9409 (80.56)	2229 (83.93)	2339 (86.94)	2408 (88.38)	2433 (89.63)	2415 (84.79)	2217 (87.49)	2355 (87.94)	2422 (89.30)	2247 (84.08)	2334 (86.76)	2408 (88.71)	2420(89.32)	2227 (82.77)	2407 (87.55)	2309 (88.16)	2466 (90.29)	2195 (83.05)	2346 (86.59)	2423 (89.54)	2445 (89.70)
*P* value	0.002	< 0.001				< 0.001				< 0.001				< 0.001				< 0.001			
Alcohol consumption, No. (%)																					
No	1471 (12.6)	431 (11.67)	409 (10.72)	330 (9.00)	301 (7.20)	425 (11.10)	360 (10.03)	378 (9.58)	308 (7.47)	423 (11.31)	437 (11.69)	368 (9.85)	243 (6.06)	483 (13.31)	369 (9.97)	266 (6.62)	353 (8.64)	387 (10.49)	386 (10.19)	364 (9.31)	334 (8.30)
Yes	10,208 (87.4)	2491 (88.33)	2511 (89.28)	2587 (91.00)	2619 (92.80)	2698 (88.90)	2374 (89.97)	2523 (90.42)	2613 (92.53)	2511(88.69)	2469(88.31)	2555 (90.15)	2673 (93.94)	2455 (86.69)	2614 (90.03)	2573 (93.38)	2566 (91.36)	2532 (89.51)	2538 (89.81)	2554 (90.69)	2584 (91.70)
*P* value	0.002	< 0.001				0.002				< 0.001				< 0.001				0.070			
BMI, No. (%), kg/m^2^																					
18.5–30.0	6957 (59.57)	1613 (54.90)	1724 (58.86)	1835 (64.30)	1785 (63.50)	1794 (57.71)	1646 (61.56)	1779 (61.33)	1738 (62.01)	1653 (56.99)	1704 (58.89)	1773 (61.89)	1827 (63.81)	1601 (54.41)	1795 (60.81)	1791 (63.72)	1770 (62.80)	1651 (56.94)	1736 (58.88)	1818 (63.51)	1752 (62.61)
< 18.5	168 (1.44)	45 (1.63)	42 (1.71)	41 (1.32)	40 (1.29)	48 (1.26)	40 (1.58)	45 (1.81)	35 (1.30)	42 (1.41)	38 (1.86)	39 (1.31)	49 (1.38)	55 (2.25)	31 (1.19)	40 (1.28)	42 (1.27)	46 (1.58)	44 (1.69)	41 (1.31)	37 (1.36)
≥ 30.0	4554 (38.99)	1264 (43.47)	1154 (39.43)	1041 (34.38)	1095 (35.20)	1281 (41.03)	1048 (36.86)	1077 (36.86)	1148 (36.70)	1239 (41.60)	1164 (39.25)	1111 (36.80)	1040 (34.82)	1282 (43.34)	1157 (38.00)	1008 (35.00)	1107 (35.93)	1222 (41.48)	1144(39.43)	1059 (35.18)	1129 (36.03)
*P* value	0.001	< 0.001				0.063				0.011				< 0.001				0.010			
History of DM, No. (%)																					
No	8467 (72.5)	2050 (76.45)	2117 (78.38)	2154 (78.95)	2146 (77.71)	2233 (76.94)	1995 (78.15)	2144 (80.26)	2095 (76.43)	2051 (75.93)	2075 (77.67)	2151 (78.72)	2190 (78.98)	2097 (77.83)	2150 (77.58)	2094 (78.80)	2126 (77.45)	2088 (78.15)	2123 (78.33)	2123 (78.12)	2133 (77.10)
Yes	3212 (27.5)	872 (23.55)	803 (21.62)	763 (21.05)	774 (22.29)	890 (23.06)	739 (21.85)	757 (19.74)	826 (23.57)	883 (24.07)	831 (22.33)	772 (21.28)	726 (21.02)	841 (22.17)	833 (22.42)	745 (21.20)	793 (22.55)	831 (21.85)	801 (21.67)	795 (21.88)	785 (22.90)
*P* value	< 0.001	0.408				0.031				0.082				0.760				0.731			
History of hypertension, No. (%)																					
No	6718 (57.52)	1607 (61.36)	1683 (64.01)	1717 (64.11)	1711 (63.56)	1702 (60.89)	1592 (63.99)	1728 (65.73)	1696 (62.86)	1646 (61.74)	1639 (62.10)	1700 (64.64)	1733 (64.40)	1721 (63.15)	1710 (64.57)	1628 (62.77)	1659 (62.85)	1648 (63.28)	1669 (63.08)	1681 (62.66)	1720 (64.17)
Yes	4961 (42.48)	1315 (38.64)	1237 (35.99)	1200 (35.89)	1209 (36.44)	1421 (39.11)	1142 (36.01)	1173 (34.27)	1225 (37.14)	1288 (38.26)	1267 (37.90)	1223 (35.36)	1183 (35.60)	1217 (36.85)	1273 (35.43)	1211 (37.23)	1260 (37.15)	1271 (36.72)	1255 (36.92)	1237 (37.34)	1198 (35.83)
*P* value	0.014	0.343				0.032				0.0743				0.644				0.820			
History of hyperlipidemia, No. (%)																					
No	3104 (26.58)	710 (25.78)	772 (28.50)	841 (30.68)	781 (29.14)	803 (27.41)	742 (29.97)	789 (29.16)	770 (28.08)	735 (26.68)	725 (27.97)	793 (28.74)	851 (30.57)	801 (27.76)	780 (29.50)	735 (27.72)	788 (29.33)	742 (27.11)	781 (29.33)	812 (29.90)	769 (27.99)
Yes	8575 (73.42)	2212 (74.22)	2148 (71.50)	2076 (69.32)	2139 (70.86)	2320 (72.59)	1992 (70.03)	2112 (70.84)	2151 (71.92)	2199 (73.32)	2181 (72.03)	2130 (71.26)	2065 (69.43)	2137 (72.24)	2203 (70.50)	2104 (72.28)	2131 (70.67)	2177 (72.89)	2143 (70.67)	2106 (70.10)	2149 (72.01)
*P* value	< 0.001	0.093				0.378				0.113				0.523				0.419			
History of congestive heart failure, No. (%)																					
No	11,352 (97.2)	2820 (97.07)	2838 (98.34)	2837 (98.29)	2857 (98.34)	3017 (97.59)	2671 (98.38)	2828 (98.30)	2836 (97.94)	2839 (97.60)	2819 (97.79)	2838 (98.12)	2856 (98.52)	2838 (97.18)	2897 (97.88)	2771 (98.72)	2846 (98.28)	2825 (97.63)	2843 (98.12)	2837 (98.06)	2847 (98.30)
Yes	327 (2.8)	102 (2.93)	82 (1.66)	80 (1.71)	63 (1.66)	106 (2.41)	63 (1.62)	73 (1.70)	85 (2.06)	95 (2.40)	87 (2.21)	85 (1.88)	60 (1.48)	100 (2.82)	86 (2.12)	68 (1.28)	73 (1.72)	94 (2.37)	81 (1.88)	81 (1.94)	71 (1.70)
*P* value	< 0.001	0.021				0.160				0.149				0.003				0.442			
History of coronary heart disease, No. (%)																					
No	11,207 (95.96)	2804 (96.20)	2811 (97.34)	2783 (96.29)	2809 (96.65)	2983 (96.37)	2635 (97.05)	2781 (96.56)	2808 (96.59)	2831 (96.86)	2775 (95.90)	2789 (96.62)	2812 (97.05)	2852 (97.21)	2863 (96.80)	2708 (96.44)	2784 (96.18)	2806 (96.62)	2816 (97.19)	2792 (96.61)	2793 (96.15)
Yes	472 (4.04)	118 (3.80)	109 (2.66)	134 (3.71)	111 (3.35)	140 (3.63)	99 (2.95)	120 (3.44)	113 (3.41)	103 (3.14)	131 (4.10)	134 (3.38)	104 (2.95)	86 (2.79)	12 0(3.20)	131 (3.56)	135 (3.82)	113 (3.38)	108 (2.81)	126 (3.39)	125 (3.85)
*P* value	< 0.001	0.183				0.722				0.204				0.043				0.271			
History of angina, No. (%)																					
No	11,390 (97.53)	2845 (97.65)	2847 (98.22)	2852 (98.13)	2846 (97.86)	3039 (97.90)	2672 (98.07)	2829 (98.10)	2850 (97.83)	2865 (97.71)	2826 (97.87)	2851 (98.23)	2848 (98.03)	2868 (97.84)	2915 (98.40)	2773 (98.04)	2834 (97.64)	2845 (97.88)	2853 (98.30)	2850 (97.93)	2842 (97.78)
Yes	289 (2.47)	77 (2.35)	73 (1.78)	65 (1.87)	74 (2.14)	84 (2.10)	62 (1.93)	72 (1.90)	71 (2.17)	69 (2.29)	80 (2.13)	72 (1.77)	68 (1.97)	70 (2.16)	68 (1.60)	66 (1.96)	85 (2.36)	74 (2.12)	71 (1.70)	68 (2.07)	76 2.22)
*P* value	< 0.001	0.548				0.091				0.681				0.414				0.671			
History of heart attack, No. (%)																					
No	11,165 (95.6)	2789 (96.57)	2783 (96.72)	2797 (96.95)	2796 (96.61)	2959 (96.28)	2637 (97.25)	2771 (96.46)	2798 (96.91)	2801 (96.75)	2780 (96.55)	2785 (96.57)	2799 (96.95)	2817 (96.90)	2860 (97.19)	2710 (96.57)	2778 (96.28)	2783 (96.65)	2793 (96.59)	2810 (97.43)	2779 (96.20)
Yes	514 (4.4)	133 (3.43)	137 (3.28)	120 (3.05)	124 (3.39)	164 (3.72)	97 (2.75)	130 (3.54)	123 (3.09)	133 (3.25)	126 (3.45)	138 (3.43)	117 (3.05)	121 (3.10)	123 (2.81)	129 (3.43)	141 (3.72)	136 (3.35)	131 (3.41)	108 (2.57)	139 (3.80)
*P* value	< 0.001	0.093				0.331				0.863				0.412				0.148			
History of stroke, No. (%)																					
No	11,206 (95.95)	2758 (95.90)	2799 (97.13)	2821 (97.85)	2828 (97.37)	2957 (96.30)	2628 (97.16)	2780 (96.67)	2841 (98.25)	2790 (96.65)	2781 (96.76)	2813 (97.22)	2822 (97.63)	2805 (97.23)	2857 (96.72)	2733 (97.47)	2811 (96.99)	2772 (96.82)	2793 (96.55)	2816 (97.55)	2825 (97.41)
Yes	473 (4.05)	164 (4.10)	121 (2.87)	96 (2.15)	92 (2.63)	166 (3.70)	106 (2.84)	121 (3.33)	80 (1.75)	144 (3.35)	125 (3.24)	110 (2.78)	94 (2.37)	133 (2.77)	126 (3.28)	106 (2.53)	108 (3.01)	147 (3.18)	131 (3.45)	102 (2.45)	93 (2.59)
*P* value	< 0.001	0.002				0.003				0.142				0.401				0.162			

*HS* high school, *BMI* body mass index.

^a^All proportions,are weighted estimates of the U.S. population characteristics, taking into account the complex sampling design of the NHANES.

### Different flavonol intake level, survival status and cause of death in participants

Table 3 presented the general characteristics of flavonol intake in participants by survival status or cause of death. As the intake of total flavonol increases, there is a decreasing trend in all-cause mortality, AD-specific mortality, cancer-specific mortality, and CVD-specific mortality (all *P* values for trend < 0.050). When isorhamnetin intake increases, there is a declining tendency in all-cause mortality, AD mortality, cancer mortality, CVD mortality, DM mortality, and other cause mortality (all *P* values for trend < 0.050). As the intake of kaempferol increases, there is a decreasing trend in all-cause mortality, AD-specific mortality, cancer-specific mortality, CVD-specific mortality, and other cause mortality (all *P* for trend < 0.050). As the intake of myricetin increases, there is a decreasing trend in AD mortality (*P* for trend = 0.003). With increasing quercetin intake quartiles, there is a trend toward lowering all-cause mortality, cancer mortality, and other cause mortality (all *P* for trend < 0.050).

**Table 3:** All-cause and specific-cause mortality characteristics by dietary flavonol intake.

Flavonol types		Study participants, (No. (%))						
		Alive	All-cause death	AD-Specific death	Cancer-Specific death	CVD-Specific Death	DM-Specific death	Other cause death
Total flavonols (mg/day)	Q1 [0, 5.81]	2522 (90.32)	400 (9.68)	15 (0.25)	112 (2.60)	131 (3.04)	12 (0.23)	130 (3.54)
	Q2 (5.81, 12.51]	2586 (92.56)	334 (7.44)	11 (0.28)	82 (1.93)	112 (2.48)	15 (0.37)	114 (2.38)
	Q3 (12.51, 23.99]	2575 (92.15)	342 (7.85)	12 (0.27)	76 (1.89)	134 (2.87)	7 (0.08)	113 (2.74)
	Q4 (23.99, 288.14]	2649 (93.84)	271 (6.16)	5 (0.11)	66 (1.34)	88 (1.96)	12 (0.26)	100 (2.50)
	*P* for trend	0.017	0.017	0.020	< 0.001	0.042	0.568	0.071
Isorhamnetin (mg/day)	Q1 [0, 0]	2675 (90.04)	448 (9.96)	18 (0.35)	110 (2.45)	143 (3.14)	15 (0.34)	162 (3.67)
	Q2 (0, 0.36]	2437 (92.79)	297 (7.21)	7 (0.14)	74 (1.75)	106 (2.49)	11 (0.23)	99 (2.59)
	Q3 (0.36, 1.13]	2592 (93.01)	309 (6.99)	9 (0.23)	75 (1.77)	113 (2.32)	15 (0.25)	97 (2.42)
	Q4 (1.13, 51.35]	2628 (93.48)	293 (6.52)	9 (0.16)	77 (1.63)	103 (2.27)	5 (0.14)	99 (2.23)
	*P* for trend	0.020	< 0.001	0.030	0.025	0.001	0.018	< 0.001
Kaempferol (mg/day)	Q1 [0, 0.53]	2546 (91.06)	388 (8.94)	18 (0.38)	101 (2.26)	127 (2.90)	14 (0.35)	128 (3.06)
	Q2 (0.53, 2.09]	2542 (90.98)	364 (9.02)	11 (0.23)	99 (2.55)	130 (3.03)	10 (0.21)	114 (3.01)
	Q3 (2.09, 6.18]	2603 (92.87)	320 (7.13)	10 (0.25)	69 (1.54)	112 (2.54)	9 (0.15)	120 (2.65)
	Q4 (6.18, 110.93]	2641 (93.88)	275 (6.12)	4 (0.08)	67 (1.43)	96 (1.93)	13 (0.25)	95 (2.45)
	*P* for trend	0.013	0.013	0.002	0.029	0.018	0.144	0.011
Myricetin (mg/day)	Q1 [0, 0.19]	2620 (92.83)	318 (7.17)	16 (0.37)	77 (1.63)	108 (2.43)	13 (0.24)	104 (2.50)
	Q2 (0.19, 0.51]	2606 (91.07)	377 (8.93)	11 (0.20)	106 (2.58)	126 (2.79)	10 (0.22)	124 (3.13)
	Q3 (0.51, 1.6]	2506 (92.45)	333 (7.55)	9 (0.20)	76 (1.87)	123 (2.73)	9 (0.23)	116 (2.51)
	Q4 (1.6, 79.76]	2600 (92.81)	319 (7.19)	7 (0.15)	77 (1.58)	108 (2.32)	14 (0.26)	113 (2.88)
	*P* for trend	0.588	0.497	0.003	0.424	0.559	0.162	0.562
Quercetin (mg/day)	Q1 [0, 3.36]	2545 (91.03)	374 (8.97)	14 (0.24)	103 (2.36)	122 (2.78)	13 (0.25)	122 (3.35)
	Q2 (3.36, 7.74]	2585 (92.64)	339 (7.36)	8 (0.19)	86 (2.06)	109 (2.25)	14 (0.29)	122 (2.57)
	Q3 (7.74, 15.07]	2554 (91.85)	364 (8.15)	15 (0.35)	74 (1.62)	148 (3.33)	9 (0.17)	118 (2.68)
	Q4 (15.07, 196.51]	2648 (93.52)	270 (6.48)	6 (0.13)	73 (1.65)	86 (1.93)	10 (0.24)	95 (2.52)
	*P* for trend	0.032	0.007	0.435	0.013	0.414	0.305	0.035

Mortality was assessed through December 31, 2019. All proportions are weighted estimates of the U.S. population characteristics, taking into account the complex sampling design of the NHANES.

*AD* Alzheimer's disease, *CVD* cardiovascular disease, *DM* diabetes mellitus.

### All-cause mortality by basic characteristics of sociodemographic variables and disease history

We applied Cox regression analysis to explore the relationship between basic characteristics (including sociodemographic variables, and disease history) and all-cause mortality in U.S. participants, and the detailed outcome was presented in Table 4. It is important to notice that HRs increased significantly every ten years of age. Female (HR 0.77; 95% CI 0.69–0.86), and Mexican American (HR 0.36; 95% CI 0.28–0.47), educational attainment ≥ high school (HR 0.49; 95% CI 0.43–0.57), being married or living with a partner (HR 0.64; 95% CI 0.56–0.72), having alcohol consumption (HR 0.60; 95% CI 0.46–0.78) were significantly related to a lower risk of all-cause mortality. What’s more, the covariates including BMI < 18.5 (HR 1.76; 95% CI 1.11–2.81), having disease history including DM (HR 2.99; 95% CI 2.55–3.51), hypertension (HR 4.25; 95% CI 3.62–5.00), hyperlipidemia (HR 4.25; 95% CI 3.62–5.00), congestive heart failure (HR 8.42; 95% CI 6.89–10.29), coronary heart disease (HR 5.35; 95% CI 4.58–6.24), angina (HR 3.70; 95% CI 2.86–4.79), heart attack (HR 4.67; 95% CI 3.85–5.67), heart attack (HR 5.54; 95% CI 4.25–7.21) were significantly related to higher risk of all-cause mortality.

**Table 4:** All-cause mortality by basic characteristics of sociodemographic variables and disease history.

Characteristic	Study participants		
	Survived	Deceased	HR (95% CI) ^a^
Age, No. (%), years			
20–29	1862 (20.52)	17 (2.31)	1 [Reference]
30–39	1916 (19.10)	26 (3.11)	1.42 (0.67, 3.02)
40–49	1933 (20.30)	70 (7.66)	3.09 (1.64, 5.82)***
50–59	1769 (19.79)	143 (13.38)	5.77 (3.21, 10.35)***
60–69	1729 (12.67)	284 (20.45)	14.23 (7.95, 25.45)***
70–79	863 (5.99)	412 (26.82)	35.30 (20.79, 59.94)***
≥ 80	260 (1.63)	395 (26.27)	94.07 (55.72, 158.81)***
Sex, No. (%)			
Male	5004 (48.67)	812 (55.42)	1 [Reference]
Female	5328 (51.33)	535 (44.58)	0.77 (0.69, 0.86)***
Race/Ethnicity, No. (%)			
Non-Hispanic White	4669 (69.44)	885 (81.36)	1 [Reference]
Non-Hispanic Black	1969 (10.04)	240 (9.67)	0.86 (0.70, 1.04)
Mexican American	1801 (8.53)	94 (3.34)	0.36 (0.28, 0.47)***
Other	1893 (11.99)	128 (5.63)	0.48 (0.35, 0.66)***
Marital status, No. (%)			
Unmarried or other	3925 (34.86)	644 (45.50)	1 [Reference]
Married or living with a partner	6407 (65.14)	703 (54.50)	0.64 (0.56, 0.72)***
Educational attainment, No. (%)			
< HS	2406 (14.01)	481 (29.67)	1 [Reference]
≥ HS	7926 (85.99)	866 (70.33)	0.49 (0.43, 0.57)***
Poverty income ratio, No. (%)			
Below poverty line (< 1.00)	1999 (12.50)	271 (14.24)	1 [Reference]
At or above poverty line (≥ 1.00)	8333 (87.50)	1076 (85.76)	0.91 (0.79, 1.06)
Alcohol consumption, No. (%)			
No	1254 (9.03)	217 (15.49)	1 [Referenc e]
Yes	9078 (90.97)	1130 (84.51)	0.60 (0.46, 0.78)***
BMI, No. (%)			
18.5–30.0	6110(60.58)	847 (61.05)	1 [Reference]
< 18.5	137 (1.40)	31 (2.39)	1.76 (1.11, 2.81)**
≥ 30.0	4085 (38.02)	469 (36.56)	1.06 (0.92, 1.22)
History of DM, No. (%)			
No	7770 (79.74)	697 (55.86)	1 [Reference]
Yes	2562 (20.26)	650 (44.14)	2.99 (2.55, 3.51)***
History of hypertension, No. (%)			
No	6348 (66.03)	370 (30.79)	1 [Reference]
Yes	3984 (33.97)	977 (69.21)	4.25 (3.62, 5.00)***
History of hyperlipidemia, No. (%)			
No	2856 (29.48)	248 (18.13)	1 [Reference]
Yes	7476 (70.52)	1099 (81.87)	1.68(1.38, 2.04)***
History of congestive heart failure, No. (%)			
No	10,159 (98.77)	1193 (89.25)	1 [Reference]
Yes	173 (1.23)	154 (10.75)	8.42 (6.89, 10.29)***
History of coronary heart disease, No. (%)			
No	10,033 (97.42)	1174 (87.12)	1 [Reference]
Yes	299 (2.58)	173 (12.88)	5.35 (4.58, 6.24)***
History of angina, No. (%)			
No	10,135 (98.33)	1255 (93.66)	1 [Reference]
Yes	197 (1.67)	92 (6.34)	3.70 (2.86, 4.79)***
History of heart attack, No. (%)			
No	10,003(97.45)	1162(87.85)	1 [Reference]
Yes	329 (2.55)	185 (12.15)	4.67 (3.85, 5.67)***
History of stroke, No. (%)			
No	10,036(97.89)	1170(87.58)	1 [Reference]
Yes	296 (2.11)	177 (12.42)	5.54 (4.25, 7.21)***

All-cause mortality was assessed through December 31, 2019. All proportions are weighted estimates of the U.S. population characteristics, taking into account the complex sampling design of the NHANES.

*HR* hazard ratio, *CI* confidence interval, *HS* high school, *BMI* body mass index, *DM* diabetes mellitus.

*P < 0.05; **P < 0.01; ***P < 0.001.

^a^Adjsuted for age and sex.

### Association between dietary flavonol intake and all-cause mortality

After adjustment for age, gender, race/ethnicity, marital status, education level, poverty income ratio, alcohol consumption, BMI, and disease history, multivariate Cox regression analysis showed a significant correlation between total flavonol intake in the highest category and all-cause mortality risk (HR 0.64, 95% CI 0.54–0.75, *P* for trend < 0.001 ), compared to the lowest quartile (Table 5). Further, we investigated the associations between intake of four types of flavonol and all-cause mortality risks. We observed that isorhamnetin (HR 0.72, 95% CI 0.60–0.86, *P* for trend < 0.001), kaempferol (HR 0.74, 95% CI 0.63–0.86, *P* for trend < 0.001), myricetin (HR 0.77, 95% CI 0.67–0.88, *P* for trend < 0.001) and quercetin (HR 0.66, 95% CI 0.54–0.81, *P* for trend < 0.001) intakes in the highest quartile were all related to all-cause mortality risks, compared to the lowest quartile (Table 5).

**Table 5:** Cox proportional hazards models for all-cause mortality and Fine and Gray competing risks regression models for specific-cause mortality by dietary flavonol intake.

Flavonol types		Due to all-cause		Due to AD		Due to cancer		Due to CVD		Due to DM		Due to other causes	
		HR (95% CI)^a^	HR (95% CI)^b^	HR (95% CI)^a^	HR (95% CI)^b^	HR (95% CI)^a^	HR (95% CI)^b^	HR (95% CI)^a^	HR (95% CI)^b^	HR (95% CI)^a^	HR (95% CI)^b^	HR (95% CI)^a^	HR (95% CI)^b^
Total flavonol (mg/day)	Q1 [0, 5.81]	1 [Reference]	1 [Reference]	1 [Reference]	1 [Reference]	1 [Reference]	1 [Reference]	1 [Reference]	1 [Reference]	1 [Reference]	1 [Reference]	1 [Reference]	1 [Reference]
	Q2 (5.81, 12.51]	0.74 (0.61, 0.91)**	0.75 (0.61, 0.92)*	1.25 (0.55, 2.81)	1.24 (0.55, 2.81)	0.67 (0.41, 1.08)	0.67 (0.41, 1.07)	0.77 (0.56, 1.07)	0.78 (0.56, 1.08)	1.81 (0.62, 5.24)	1.78 (0.61, 5.18)	0.63 (0.48, 0.81)***	0.63 (0.49, 0.81)***
	Q3 (12.51, 23.99]	0.80 (0.68, 0.95)*	0.82 (0.69, 0.96)*	1.64 (0.68, 3.99)	1.65 (0.68, 3.97)	0.70 (0.44, 1.12)	0.69 (0.44, 1.10)	0.97 (0.72, 1.32)	0.98 (0.72, 1.33)	0.48 (0.12, 1.90)	0.46 (0.12, 1.85)	0.74 (0.54, 1.00)*	0.73 (0.54, 0.99)*
	Q4 (23.99, 288.14]	0.64 (0.54, 0.75)***	0.66 (0.56, 0.77)***	0.76 (0.27, 2.13)	0.77 (0.28, 2.14)	0.45 (0.28, 0.70)***	0.44 (0.28, 0.69)***	0.67 (0.47, 0.96)*	0.69 (0.48, 0.98)*	1.44 (0.45, 4.59)	1.38 (0.43, 4.39)	0.64 (0.51, 0.81)***	0.60 (0.48, 0.75)***
	*P* for trend	< 0.001	< 0.001	0.940	0.970	0.003	0.002	0.080	0.010	0.980	0.960	0.003	< 0.001
Isorhamnetin (mg/day)	Q1 [0, 0]	1 [Reference]	1 [Reference]	1 [Reference]	1 [Reference]	1 [Reference]	1 [Reference]	1 [Reference]	1 [Reference]	1 [Reference]	1 [Reference]	1 [Reference]	1 [Reference]
	Q2 (0, 0.36]	0.70 (0.59, 0.84)***	0.71 (0.59, 0.84)***	0.42 (0.11, 1.59)	0.42 (0.11, 1.60)	0.65 (0.47, 0.89)*	0.64 (0.47, 0.89)*	0.77 (0.56, 1.05)	0.77 (0.56, 1.05)	0.67 (0.24, 1.88)	0.66 (0.24, 1.83)	0.66 (0.46, 0.94)*	0.67 (0.47, 0.96)*
	Q3 (0.36, 1.13]	0.73 (0.61, 0.88)**	0.74 (0.61, 0.89)**	0.84 (0.37, 1.89)	0.85 (0.38, 1.89)	0.67 (0.46, 0.98)*	0.67 (0.46, 0.97)*	0.77 (0.55, 1.07)	0.77 (0.55, 1.08)	0.82 (0.33, 2.04)	0.80 (0.32, 1.99)	0.68 (0.51, 0.90)*	0.70 (0.52, 0.93)*
	Q4 (1.13, 51.35]	0.72 (0.60, 0.86)***	0.73 (0.61, 0.88)***	0.74 (0.22, 2.51)	0.75( 0.22, 2.52)	0.62 (0.46, 0.83)**	0.61 (0.46, 0.82)**	0.81 (0.58, 1.13)	0.82 (0.58, 1.15)	0.48 (0.14, 1.67)	0.47 (0.13, 1.63)	0.66 (0.47, 0.92)*	0.71 (0.50, 0.92)*
	*P* for trend	< 0.001	< 0.001	0.710	0.720	0.010	0.004	0.210	0.240	0.300	0.280	0.010	0.040
Kaempferol (mg/day)	Q1 [0, 0.53]	1 [Reference]	1 [Reference]	1 [Reference]	1 [Reference]	1 [Reference]	1 [Reference]	1 [Reference]	1 [Reference]	1 [Reference]	1 [Reference]	1 [Reference]	1 [Reference]
	Q2 (0.53, 2.09]	0.81 (0.69, 0.94)*	0.80 (0.69, 0.94)*	0.47 (0.23, 0.98)*	0.47 (0.23, 0.97)*	0.89 (0.65, 1.23)	0.89 (0.65, 1.23)	0.80 (0.61, 1.04)	0.80 (0.61, 1.03)	0.56 (0.17, 1.83)	0.56 (0.17, 1.86)	0.86 (0.65, 1.13)	0.85 (0.65, 1.11)
	Q3 (2.09, 6.18]	0.69 (0.56, 0.85)***	0.70 (0.57, 0.85)***	0.69 (0.23, 2.02)	0.69 (0.23, 2.03)	0.59 (0.36, 0.95)*	0.59 (0.36, 0.95)*	0.76 (0.55, 1.05)	0.76 (0.55, 1.06)	0.47 (0.16, 1.44)	0.47 (0.15, 1.43)	0.80 (0.56, 1.15)	0.82 (0.58, 1.18)
	Q4 (6.18, 110.93]	0.74 (0.63, 0.86)***	0.76 (0.65, 0.88)***	0.37 (0.12, 1.11)	0.37 (0.12, 1.12)	0.62 (0.40, 0.97)*	0.62 (0.40, 0.95)*	0.73 (0.53, 1.00)	0.74 (0.54, 1.02)	0.93 (0.28, 3.10)	0.90 (0.27, 3.01)	0.86 (0.64, 1.15)	0.94 (0.70, 1.26)
	*P* for trend	< 0.001	< 0.001	0.160	0.170	0.020	0.010	0.060	0.080	0.870	0.830	0.270	0.660
Myricetin (mg/day)	Q1 [0, 0.19]	1 [Reference]	1 [Reference]	1 [Reference]	1 [Reference]	1 [Reference]	1 [Reference]	1 [Reference]	1 [Reference]	1 [Reference]	1 [Reference]	1 [Reference]	1 [Reference]
	Q2 (0.19, 0.51]	0.92 (0.76, 1.11)	0.93 (0.77, 1.12)	0.36 (0.13, 0.97)*	0.36 (0.13, 0.98)*	1.06 (0.78, 1.44)	1.05 (0.78, 1.43)	0.71 (0.51, 1.00)*	0.73 (0.53, 1.00)*	0.84 (0.34, 2.04)	0.83 (0.34, 2.04)	0.87 (0.63, 1.21)	0.91 (0.66, 1.25)
	Q3 (0.51, 1.6]	0.74 (0.63, 0.87)***	0.76 (0.64, 0.90)**	0.43 (0.13, 1.35)	0.43 (0.14, 1.36)	0.74 (0.48, 1.13)	0.72 (0.47, 1.12)	0.68 (0.51, 0.92)*	0.67 (0.49, 0.92)*	0.93 (0.26, 3.36)	0.90 (0.25, 3.26)	0.62 (0.44, 0.86)**	0.64 (0.46, 0.89)*
	Q4 (1.6, 79.76]	0.77 (0.67, 0.88)***	0.79 (0.69, 0.91)***	0.34 (0.14, 0.85)*	0.35 (0.14, 0.87)*	0.65 (0.42, 0.99)*	0.64 (0.42, 0.98)*	0.61 (0.47, 0.80)***	0.62 (0.46, 0.83)**	1.07 (0.38, 3.01)	1.03 (0.37, 2.83)	0.70 (0.53, 0.91)*	0.74 (0.57, 0.97)*
	*P* for trend	< 0.001	< 0.001	0.090	0.090	0.010	0.010	< 0.001	0.003	0.830	0.890	0.010	0.020
Quercetin (mg/day)	Q1 [0, 3.36]	1 [Reference]	1 [Reference]	1 [Reference]	1 [Reference]	1 [Reference]	1 [Reference]	1 [Reference]	1 [Reference]	1 [Reference]	1 [Reference]	1 [Reference]	1 [Reference]
	Q2 (3.36, 7.74]	0.72 (0.61, 0.85)***	0.73 (0.62, 0.86)***	0.71 (0.32, 1.54)	0.71 (0.33, 1.54)	0.74 (0.52, 1.05)	0.73 (0.52, 1.05)	0.67 (0.50, 0.90)*	0.67 (0.50, 0.90)*	1.22 (0.50, 2.95)	1.21 (0.50, 2.92)	0.62 (0.48, 0.81)***	0.66 (0.51, 0.87)**
	Q3 (7.74, 15.07]	0.84 (0.71, 0.98)*	0.85 (0.72, 0.99)*	1.85 (0.72, 4.73)	1.85 (0.73, 4.72)	0.62 (0.39, 0.98)*	0.61 (0.39, 0.96)*	1.08 (0.83, 1.40)	1.09 (0.83, 1.41)	0.84 (0.19, 3.58)	0.81 (0.19, 3.49)	0.69 (0.52, 0.91)*	0.72 (0.54, 0.96)*
	Q4 (15.07, 196.51]	0.66 (0.54, 0.81)***	0.66 (0.54, 0.82)***	0.73 (0.30, 1.82)	0.74 (0.30, 1.84)	0.54 (0.35, 0.84)*	0.53 (0.35, 0.82)**	0.61 (0.40, 0.93)*	0.62 (0.41, 0.94)*	1.11 (0.38, 3.24)	1.07 (0.37, 3.09)	0.61 (0.46, 0.80)***	0.62 (0.45, 0.85)**
	*P* for trend	< 0.001	< 0.001	0.750	0.730	0.004	0.003	0.130	0.160	0.970	0.920	< 0.001	0.005

*HR* hazard ratio, *CI* confidence interval, *AD* Alzheimer's disease, *CVD* cardiovascular disease, *DM* diabetes mellitus.

*P < 0.05; **P < 0.01; ***P < 0.001.

^a^Adjusted for age, sex, race/ethnicity, educational attainment, marital status, poverty income ratio, alcohol consumption, BMI, disease history (DM, hypertension, hyperlipidemia, congestive heart failure, coronary heart disease, angina, heart attack, stroke).

^b^Adjusted for age, squared age, sex, race/ethnicity, educational attainment, marital status, poverty income ratio, alcohol consumption, BMI, disease history (DM, hypertension, hyperlipidemia, congestive heart failure, coronary heart disease, angina, heart attack, stroke).

### Association between dietary flavonol intake and cause-specific mortality

Of the 11,679 subjects in this study, 43 (0.03%) died of AD, 336 (2.88%) died of cancer, 486 (4.16%) died of CVD, 46 (0.40%) died of DM, and 457 (3.91%) died from other causes. After adjusting covariates, the multivariate cox regression analysis showed that total flavonol intake in the highest quartile was significantly associated with lower cancer-specific mortality (HR 0.45, 95% CI 0.28–0.70, *P* for trend = 0.003), CVD-specific mortality (HR 0.67, 95% CI 0.47–0.96, *P* for trend = 0.080) and other cause mortality (HR 0.64, 95% CI 0.51–0.81, *P* for trend = 0.003), compared to the lowest quartile; isorhamnetin intake in the highest quartile was significantly related to lower cancer-specific mortality (HR 0.62, 95% CI 0.46–0.83, *P* for trend = 0.010), and other cause mortality (Q4 vs. Q1, HR 0.66, 95% CI 0.47–0.92, *P* for trend = 0.010), compared to the lowest quartile; kaempferol intake in the highest category was significantly related to lower cancer-specific mortality (HR 0.62, 95% CI 0.40–0.97, *P* for trend = 0.020), compared to the lowest category; myricetin intake in the highest category was significantly related to lower AD-specific mortality risk (HR 0.34, 95% CI 0.14–0.85, *P* for trend = 0.090), cancer-specific mortality risk (HR 0.65, 95% CI 0.42–0.99, *P* for trend = 0.010), CVD-specific mortality risk (HR 0.61, 95% CI 0.47–0.80, *P* for trend < 0.001), and other cause mortality risk (HR 0.70, 95% CI 0.53–0.91, *P* for trend = 0.010), compared to the lowest category; quercetin intake in the highest quartile was significantly associated with lower cancer-specific mortality risk (HR 0.54, 95% CI 0.35–0.84, *P* for trend = 0.004), CVD-specific mortality risk (HR 0.61, 95% CI 0.40–0.93, *P* for trend = 0.130), and other cause mortality risk (HR 0.61, 95% CI 0.46–0.80, *P* for trend < 0.001), compared to the lowest quartile (Table 5).

### Subgroup analysis stratified by race/ethnicity

After adjustment for age, gender, race/ethnicity, marital status, education level, poverty income ratio, alcohol consumption, BMI, and disease history, the subgroup analysis stratified by race/ethnicity showed that total flavonol (HR 0.59, 95% CI 0.49–0.72, *P* for trend < 0.001), isorhaminetin (HR 0.63, 95% CI 0.52–0.77, *P* for trend < 0.001), kaempferol (HR 0.62, 95% CI 0.50–0.76, *P* for trend < 0.001), myricetin (HR 0.97, 95% CI 0.81–1.17, *P* for trend < 0.001) and quercetin (HR 0.68, 95% CI 0.55–0.83, *P* for trend < 0.001) intake in highest quartile were significantly associatied with lower all-cause mortality risk in Non-Hispanic White, compared with lowest quartile. More detailed outcomes were presented in Table 6. Compared to Non Hispanic Black, Mexican American, and other race/enthnicity, dietary flavonol intake has a more significant protective effect on the Non Hispanic White population.

**Table 6:** Subgroup analysis stratified by race/ethnicity, using Cox proportional hazards models for all-cause mortality by dietary flavonol intake.

Flavonol types		Non-Hispanic White	Non-Hispanic Black	Mexican American	Other
		HR (95% CI)^a^	HR (95% CI)^a^	HR (95% CI)^a^	HR (95% CI)^a^
Total flavonols (mg/day)	Q1 [0, 5.81]	1 [Reference]	1 [Reference]	1 [Reference]	1 [Reference]
	Q2 (5.81, 12.51]	0.79 (0.63, 0.98)*	0.66 (0.41, 1.07)	0.79 (0.41, 1.50)	0.45 (0.20, 0.98)*
	Q3 (12.51, 23.99]	0.77 (0.59, 1.02)	0.96 (0.68, 1.36)	0.86 (0.51, 1.45)	0.84 (0.39, 1.81)
	Q4 (23.99, 288.14]	0.59 (0.49, 0.72)***	0.75 (0.46, 1.20)	0.69 (0.32, 1.51)	0.46 (0.26, 0.84)*
	*P* for trend	< 0.001	0.456	0.390	0.121
Isorhamnetin (mg/day)	Q1 [0, 0]	1 [Reference]	1 [Reference]	1 [Reference]	1 [Reference]
	Q2 (0, 0.36]	0.70 (0.57, 0.86)*	0.59 (0.40, 0.88)*	0.84 (0.54, 1.31)	1.02 (0.40, 2.60)
	Q3 (0.36, 1.13]	0.70 (0.55, 0.89)**	0.79 (0.49, 1.28)	0.84 (0.48, 1.48)	0.53 (0.26, 1.08)
	Q4 (1.13, 51.35]	0.63 (0.52, 0.77)***	0.78 (0.50, 1.20)	0.62 (0.33, 1.16)	0.85 (0.41, 1.77)
	*P* for trend	< 0.001	0.318	0.160	0.438
Kaempferol (mg/day)	Q1 [0, 0.53]	1 [Reference]	1 [Reference]	1 [Reference]	1 [Reference]
	Q2 (0.53, 2.09]	1.113 (0.90, 1.38)	0.84 (0.514, 1.39)	0.65 (0.38, 1.09)	0.64 (0.30, 1.38)
	Q3 (2.09, 6.18]	0.810 (0.66, 0.99)*	0.69 (0.421, 1.12)	0.60 (0.29, 1.22)	0.67 (0.26, 1.74)
	Q4 (6.18, 110.93]	0.618 (0.50, 0.76)***	1.05 (0.632, 1.76)	0.69 (0.30, 1.56)	0.49 (0.29, 0.82)**
	*P* for trend	< 0.001	0.88	0.368	0.052
Myricetin (mg/day)	Q1 [0, 0.19]	1 [Reference]	1 [Reference]	1 [Reference]	1 [Reference]
	Q2 (0.19, 0.51]	1.31 (0.99, 1.71)	1.25 (0.92, 1.69)	1.24 (0.65, 2.36)	0.88 (0.38, 2.03)
	Q3 (0.51, 1.6]	1.03 (0.81, 1.30)	0.98 (0.656, 1.459)	1.31 (0.62, 2.78)	1.01 (0.44, 2.30)
	Q4 (1.6, 79.76]	0.97 (0.81, 1.17)	0.82 (0.443, 1.508)	1.93 (0.77, 4.80)	0.61 (0.34, 1.08)
	*P* for trend	0.212	0.452	0.148	0.188
Quercetin (mg/day)	Q1 [0, 3.36]	1 [Reference]	1 [Reference]	1 [Reference]	1 [Reference]
	Q2 (3.36, 7.74]	0.80 (0.66, 0.96)*	0.69 (0.48, 0.99)*	1.36 (0.71, 2.61)	0.66 (0.35, 1.24)
	Q3 (7.74, 15.07]	0.89 (0.72, 1.11)	1.10 (0.72, 1.69)	0.84 (0.49, 1.43)	0.77 (0.47, 1.27)
	Q4 (15.07, 196.51]	0.68 (0.55, 0.83)***	0.54 (0.33, 0.88)*	0.84 (0.33, 2.10)	0.96 (0.43, 2.16)
	*P* for trend	< 0.001	0.016	0.694	0.920

*HR* hazard ratio, *CI* confidence interval.

*P < 0.05; **P < 0.01; ***P < 0.001.

^a^Adjusted for age, sex, educational attainment, marital status, poverty income ratio, alcohol consumption, BMI, disease history (DM, hypertension, hyperlipidemia, congestive heart failure, coronary heart disease, angina, heart attack, stroke).

### Sensitivity analysis

In our sensitivity analysis, we applied the final model (Cox proportional risk regression model and Fine and Gray competing risk regression model) including squared age in order to adjust the potential non-linear association between age and mortality. The observed results were consistent with those shown in the main analysis, which indicated our results were relatively robust (Table 5).

## Discussion

Established the up-to-date NHANES follow-up data and NDI mortality data, we evaluated a nationally representative sample of 11,679 U.S. citizens aged 20 years old and above, representing 44,189,187 non-institutionalized U.S. residents, and we observed that flavonol intake was significantly related to lower all-cause mortality risk. What’s more, intakes of total flavonol, isorhamnetin, kaempferol and quercetin were associated with lower cancer-specific mortality risk. Meanwhile, intakes of total flavonol, quercetin and myricetin were associated with reduced CVD-specific mortality risk. We also observed that myricetin intake was related to decreased mortality risk for AD-specific causes. As a result, consumption of flavonoids is protective against all cause, cancer, and CVD-mortality in particular. Subgroup analysis based on age further revealed that flavonoid intake had a more significant protective effect on all-cause mortality reduction in those aged ≥ 40 years compared to participants aged < 40 years.

Compared to 2020, the global burden of cancer is estimated to increase by 47% in 2040, from 19.3 million cases to 28.4 million cases. Approximately one-third of cancer cases in affluent nations are linked to physical activity, nutrition, and food. It has been suggested that consuming an appropriate amount of fruits and vegetables high in flavonoids helps prevent cancer^17^. This study suggests an inverse association between flavonol intake and cancer mortality, which is consistent with the conclusions of previous epidemiological studies by Bondonno et al.^18^ and Zhou et al.^19^. The previous meta-analysis also highlighted the negative relationship between flavonoid intake and the incidence rate of some cancers, including breast cancer^20^, prostate cancer^21^, lung cancer, gastric cancer and colorectal cancer^22^, and cancers related to smoking^22^. There is evidence that flavonol inhibit to the development and spread of cancer in vitro. For example, by focusing on the phosphoinositide 3-kinase / protein kinase B signaling pathway, kaempferol can suppress epithelial mesenchymal transition and cause apoptosis and cell cycle arrest in the G2 / M phase^23^. By targeting cell cycle proteins, quercetin causes cell cycle arrest in the G1 phase^24^, which prevents cells from proliferating. Furthermore, a high quercetin content can prevent the cell cycle from moving from G0/G1 to G2/M^25^. Quercetin has an impact on the cell cycle, but it can also cause apoptosis by signaling the pro-apoptotic PI3K/Akt and mitogen-activated protein kinase pathways^26,27^. Similar to other flavonol, however, it should be noted that flavonoids can exhibit toxic effects at high doses^28,29^. After the phenolic ring containing flavonoids is oxidized by oxidative enzymes, cytotoxic phenoxyl radicals are generated; unsaturated lipids, nucleic acids, ascorbate, nicotinamide, adenine dinucleotide, and glutathione are all oxidized by CO, which produces reactive oxygen species and is harmful to mitochondria. In some circumstances, quercetin and other flavonoids have been shown to cause a notable frequency of sister chromatid swaps and micronuclei as well as to suppress cell growth by their pro-oxidant properties^30^. Thus, it is necessary to look at how dietary supplements containing flavonoids affect cancer survival.

It is noteworthy that, following numerous confounding factor adjustments, we discovered that the highest flavonoid intake was linked to a lower risk of CVD death in comparison to the lowest consumption. This outcome is in line with the findings of a previous meta-analysis performed by Huxley et al., which revealed that there may be a negative correlation between the death rate from coronary heart disease and flavonol intake^31^. In a meta-analysis including sixteen studies, Wang et al. found that a 20 mg/d increase in flavonol consumption was linked to a 14% reduction in the risk of stroke (RR, 0.86; 95% CI 0.77–0.96)^32^. The possible mechanisms by which flavonol reduce the risk of CVD may involve multiple pathways, which have been reported to be generally associated with their Vasodilatory, anti-inflammatory, and antioxidant properties^33,34^. Using flavonol as an example, a large body of research indicates that quercetin and other related flavonol may provide protection against the most prevalent CVD. By preventing one or more processes linked to the development of the disease, such as oxidative stress, endothelial dysfunction, and inflammation, flavonol protects against atherosclerosis^3^. By reducing endothelial dysfunction, hypertension, and atherosclerosis, flavonol can protect coronary arteries^3^. The majority of acute coronary events are caused by myocardial ischemia following the rupture of atherosclerotic plaque. On the other hand, quercetin can stabilize atherosclerotic plaques by decreasing matrix metalloproteinase expression^35^. Flavonoids may affect stroke at different stages. Flavonol can decrease excitatory toxicity, regulate oxidative stress, stop platelet aggregation and thrombosis, and enhance cerebral blood flow during the acute phase^36^. In the intermediate stage, flavonol can protect the integrity of endothelial cells and reduce inflammatory responses^37,38^. Flavonol may obstruct ischemia-induced cell death pathways such as necrosis and apoptosis in later stages^39–41^.

DM remains one of the major healthcare challenges in the world. Although there is a growing understanding of the pathophysiology of diabetes, currently available treatment methods can only provide a temporary hypoglycemic effect, and cannot completely prevent the development of these abnormalities^42^. Previous literature has reported that flavonol has the potential of anti- diabetes. For example, Yang et al. reported that kaempferol can regulate lipid metabolism, improve insulin resistance, improve insulin signal transduction, and restore the imbalance between autophagy and apoptosis to protect β cells^43^. Kalai et al. reviewed that isorhamnetin can lower the impact of diabetes related diseases by reducing glucose level, improving oxidation status, reducing inflammation, and regulating lipid metabolism and adipocyte differentiation^44^. Li et al. reported that long-term intake of myricetin can inhibit apoptosis of pancreatic islets β cells and regulation of glucose levels, and myricetin can induce glucose dependent insulin secretion^45^. According to Eid et al., quercetin regulates systemic glucose homeostasis by interacting with numerous molecular targets in the liver, skeletal muscle, adipose tissue, small intestine, and pancreas^46^. The anti -diabetes mechanism of quercetin involves inhibition of intestinal glucose absorption, insulin secretion, insulin sensitizing activity, and improvement of glucose utilization in peripheral tissues. However, our results did not indicate significant correlations between flavonol intake and DM-specific mortality (all *P* values > 0.05). Whether the flavonol intake can prevent the progress of diabetes and protect diabetic patients still needs to be further explored by longitudinal studies in the population.

Alzheimer's disease (AD) is one of the most prevalent types of age-related neurodegenerative illness. There is an urgent need for effective treatment and prevention strategies since the present therapy options are not able to appreciably modify the development of AD. Myricetin was related to a lower risk of AD-specific mortality in this investigation. Myricetin has been shown to be helpful in treating Alzheimer's disease (AD) in animal trials. Ramezani et al. showed that intraperitoneal injection of myricetin dramatically raised the number of hippocampal CA3 pyramidal neurons in Alzheimer's disease rats, improving learning and memory problems^47^. Myricetin treatment dramatically reversed the novel effects of polyamines in the AD mouse model, which included downregulating brain iron and inhibiting acetylcholinesterase (AChE). Furthermore, myricetin administration improves the activity of antioxidant enzymes and decreases oxidative damage in mice^45^. In summary, our research indicates that consuming more myricetin may help stop or slow the progression of AD.

Previous studies did not take into account the competing risks of death when calculating specific causes of death. In our approach, we tackled this methodological challenge by employing a multiple confounder-adjusted competing risks model. This allowed us to elucidate the significant association between dietary flavonol intake and all-cause and specific-cause mortality risks. Nevertheless, the study still includes the following limitations: first, more than half of the sample size lacks data on dietary flavonol intake in the NHANES database, and the remaining 11,679 participants may not be nationally representative. Second, only an estimate of the flavonol dose was made at baseline, therefore it may not precisely reflect the flavonol intake over the course of the investigation. Third, the NHANES collected the representative participants in the North America, the western dietary pattern is most prevalent, which has an impact on the dietary composition of flavonol and other nutrients. Due to the limitation of database, we were unable to give the main food sources for dietary flavonol intake. Further, subgroup analysis showed that flavonol intake had a significant protective effect on mortality in the Non-Hispanic White population, suggesting that dietary patterns of different race/ethnicity may play an important role. Fourth, since the adjusted confounding factors in the model do not include total energy intake, micronutrient/antioxidant supplement intake, vitamin intake, and other dietary factors like lycopene or green tea catechins, it may be impossible to exclude the influence of these factors on the observed effect of flavonol intake.

## Conclusion

Through comprehensive updating NHANES records, this study concluded that dietary flavonol intake was significantly linked with overall, AD, cancer, and CVD-specific mortality risks. The outcome of our research elucidated the relationship between flavonol intake, all-cause, and cause-specific mortality risks in a sample representing the entire nation of non-hospitalized citizens in the United States, presenting evidence for flavonol intake as an independent, practical, quantitative, and reliable predictor of disease survival status, this means that it is suitable for the health and risk alert management of AD, CVD and cancer patients. Our findings have practical significance for public health, because flavonol can be supplemented by making daily dietary modifications and eating habits better.

## Methods

### Study population

**Figure 1:**
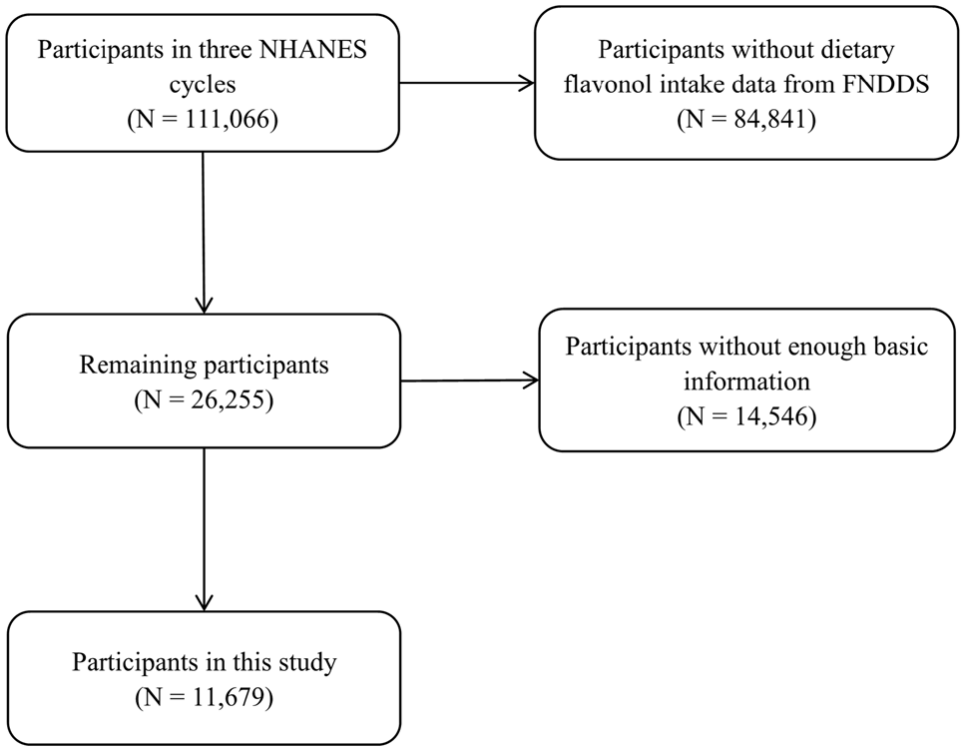
The flowchart of participants in NHANES 2007–2018.

Dietary Flavonol intake data in this study were collected from three cycles of the nationwide representative NHANES, corresponding to years 2007–2008, 2009–2010, and 2017–2018. The National Center for Health Statistics, which examined the health and nutritional status of U.S. citizens using a nationally representative sample, sponsored and supervised NHANES^22^. The three cycles NHANES provided a total of 111,066 participants. 84,841 participants were removed because they did not have dietary flavonol intake values from FNDDS, the percentage of missing values for dietary flavonol intake were 76.4%. Additionally, 14,546 individuals were excluded because they were who had no data on marital status, educational attainment, poverty income ratio, alcohol consumption, and body mass index (BMI), and disease history (Fig. 1). Finally, the present analyses were based on a total of 11,679 individuals who were aged ≥ 20 y and had completed a battery of questionnaires, in-person assessments, and laboratory tests were mandated for participants, either at the mobile examination center (MEC) or at home. The National Center for Health Statistics Research Ethics Review Board approved the NHANES programs, and all study participants gave written informed consent and followed the guidelines of the Helsinki's Declaration^48^. Relevant ethical certifications can be found in Supplement A.

### Dietary flavonol intake assessment

The purpose of conducting a dietary interview through the 24-h recall method is to gather comprehensive information about the dietary intake of NHANES participants . This method involves obtaining detailed data on the types and quantities of foods and beverages (including all forms of water) consumed in the 24-h period preceding the interview (midnight to midnight). The collected information is then used to estimate nutrient intake and other food components from the reported foods and beverages. Each NHANES participant is eligible for two 24-h dietary recall interviews, with the first interview conducted in-person at the Mobile Examination Center (MEC), and the second interview conducted via telephone 3 to 10 days later. In our study, the data on dietary flavonol intake were sourced from the USDA Survey Food and Beverage Flavonoid Values database established in 2003–2004^49^. This database contains flavonol values for various foods and beverages listed in the USDA Food and Nutrient Database for Dietary Studies (FNDDS), along with corresponding dietary data from NHANES^50^. The specific amounts of total flavonols, including isohamnetin, kaempferol, myricetin, and quercetin, measured in milligrams per 100 g, were determined for each food and beverage by the USDA Nutrient Data Laboratory^51^. The calculation of dietary flavonol intake was performed on a daily basis.

### Mortality data

For this investigation, the National Death Index (NDI) file and the 2019 Public Access Link mortality dataset were utilized to generate mortality data using a probabilistic matching technique^52^. The personal identification information of all NHANES participants aged 18 and above, including name, gender, date of birth, etc., is matched between the NHANES and NDI databases. Codes C00-C97 were designated as cancer-specific causes of death under the International Statistical Classification of Diseases and Related Health Problems, Tenth Revision (ICD-10). Causes of mortality directly connected to cardiovascular disease (CVD) were identified as heart disease (ICD-10: I00-I09, I11, I13, I20-I51), and cerebrovascular disease (ICD-10: I60-I69). Codes E10-E14 in were used to identify the causes of mortality that were primarily related to diabetes mellitus (DM). Code G30 in ICD-10 was used to identify the cause of mortality that associated with Alzheimer's disease (AD). Other causes of death were considered for the remaining fatalities that were not categorized. The follow-up period was determined by taking time from the date of the baseline interview and death or the end date of the study review, which is December 31, 2019, whichever comes first.

### Covariates

In this study, participants from NHANES were stratified by sociodemographic variables and disease history. Age was grouped as: 20–29 y, 30–39 y, 40–49 y, 50–59 y, 60–69 y, 70–79 y, and ≥ 80 y. Sex was coded as male or female. Race/ethnicity was classified as: non-Hispanic white, non-Hispanic black, Mexican American, other Hispanic, and other. Marital status was coded as: unmarried or other, married or living with a partner. Education level was coded as lower than high school (< HS), at or higher than high school (≥ HS). Poverty ratio was grouped as: below poverty line (< 1.00), at or above poverty line (≥ 1.00). Alcohol consumption was classified as: no, and yes. BMI, calculated as weight in kilograms divided by height in meters squared, was grouped as: < 18.5, 18.5–30.0, ≥ 30.0 kg/m^2^. Disease history (yes/no) included DM, hypertension, hyperlipidemia, congestive heart failure, coronary heart disease, angina, heart attack, and stroke.

### Statistical analysis

Three NHANES cycles (2007–2008, 2009–2010, and 2017–2018) were integrated into one dataset, and all analyses were carried out using the design that the NHANES Analytics and Reporting Guide supplied. The intake of dietary flavonol including total flavonol, isorhamnetin, kaempferol, myricetin, and quercetin, was classified by quartiles, with quartile 1 (Q1) indicating the lowest intake, and quartile 4 (Q4) indicating the highest intake. The basic characteristics of each participant and their dietary flavonol intake distribution were described using numbers and weighted percentages for included categorical variables. These comprised the following: age, sex, race/ethnicity, marital status, educational attainment, poverty income ratio, alcohol consumption, and BMI. A chi-square test was applied for categorical variables. Then, we described the number and percentage of overall deaths and specific-cause deaths, corresponding to different intake levels of flavonol. And trend chi-square test was applied to determine the relationship between dietary flavonol intake and death outcomes in participants. To determine basic characteristics (sociodemographic variables and disease history) related to survival endpoints, we performed the Cox regression analysis adjusted age and sex to estimate the hazard ratio (HR) and 95% confidence interval (CI) for survival. Further, covariates significantly correlated with mortality and dietary flavonol intake were added to the Cox regression model, which calculated the HR and population attributed risk of dietary flavonol intake on mortality. We applied the Fine and Gray competing risks regression model to estimate specific-cause (e.g., AD, cancer, CVD, DM) mortality risks, given that deaths from other causes can be considered the competing risk event for one specific cause of death^53^, and this method has been used by previous studies with high quality^54,55^. Sensitivity analyses was perform by adjusting for age and squared age to evaluate the potential non-linear association between age and mortality. The Variance Inflation Factor (VIF) was used to examine the covariate effect, and all VIFs were less than 3.00. The P-values on both sides are less than 0.05, which is considered statistically significant. R software (version 4.2.1, http://www.R-project.org) was applied to analyze all the data, with packages "nhanesR", "reshape2 ", "survey", "do", "dplyr" and others.

### Ethics statement

These studies involving humans have been approved by the Ethics Review Board of the National Center for Health Statistics. The studies were conducted in accordance with local legislation and institutional requirements. According to national legislation and institutional requirements, participants or their legal guardians/next of kin do not require written informed consent.

### Supplementary Information

Supplementary Information.

## Data Availability

The datasets generated during the current study are freely available without restriction in the NHANES repository (http://www.cdc.gov/nchs/nhanes.htm).
