# Supplementary material for: Association between dietary flavonol intake and mortality risk in the U.S. adults from NHANES database
Source: Sci Rep. 2024 Feb 25;14:4572. doi: 10.1038/s41598-024-55145-y (PMC10894877; doi:10.1038/s41598-024-55145-y)

**Supplement Material A**

**Association of Dietary Flavonol Intake with Risk of All-Cause and Cause-Specific Mortality: Analyses of NHANES 2007-2018 Data**

Zhiqiang Zong^a,†^, Xiang Cheng^a,†^, Jianchao Qiao^b^, Yang Yang^a^, Fanfan Li^a, *^

^†^ These authors contributed equally: Zhiqiang Zong, Xiang Cheng.

^*^ Correspondence: [fflahykdx@163.com](mailto:Yunquanzhang@wust.edu.cn) (F.F.L).


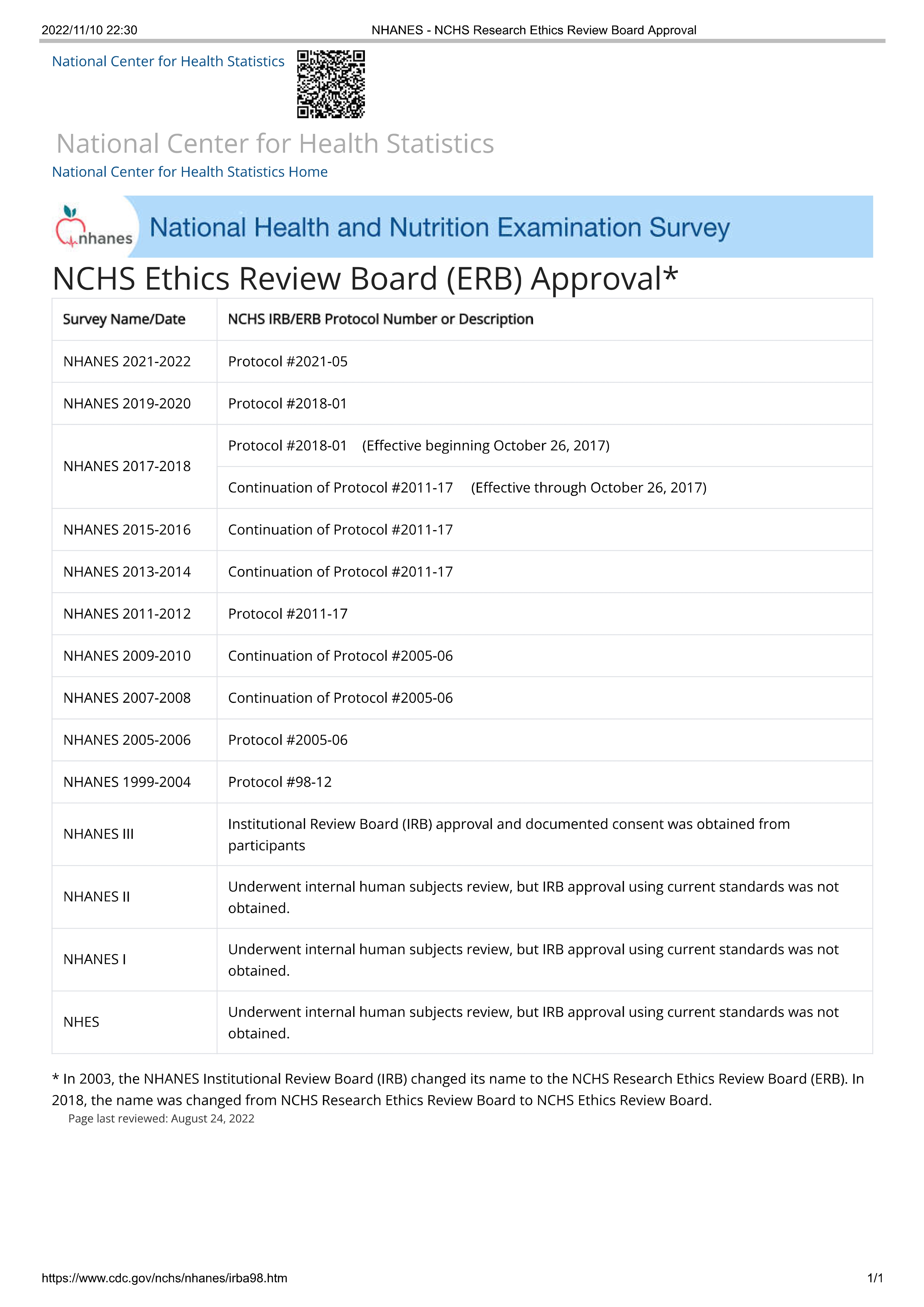

Supplement: Supplementary file 1 — Supplementary Information. [file 41598_2024_55145_MOESM1_ESM.docx]
